# Do Leaf Temperatures Determine Endophyte Composition and Richness in Florida Native Plant Species?

**DOI:** 10.1002/ece3.74615

**Published:** 2026-11-06

**Authors:** Olga Tserej, Damian J. Hernandez, Amanda H. Rawstern, Kasey N. Kiesewetter, Brianna K. Almeida, Michelle E. Afkhami, Kenneth J. Feeley

**Affiliations:** ^1^ Biology Department University of Miami Coral Gables Florida USA; ^2^ BLUE Missions Miami Florida USA; ^3^ University of Toronto Toronto Canada; ^4^ University of Waikato Hamilton New Zealand; ^5^ North Carolina State University Raleigh North Carolina USA

**Keywords:** climate change, experimental warming, fungal community composition, fungal endophytes, leaf temperature, subtropical plants

## Abstract

Microbiomes play crucial roles in ecosystems, largely through their interactions with plants. Fungal endophytes occur in nearly all plant tissues and can influence host physiology, stress tolerance, and productivity. Although previous studies have examined how air temperature shapes foliar endophyte communities, endophytes may respond more strongly to leaf temperatures, which are often decoupled from ambient air temperatures. Consequently, changes in leaf temperature (e.g., due to global warming) may alter endophyte diversity and composition. In this study, we experimentally tested whether elevated leaf temperatures affect fungal endophyte richness and community composition in six native subtropical plant species from South Florida. Using in situ localized heating of individual branches, we increased leaf temperatures by 1°C–3°C over a 2‐week period. Leaf thermal profiles were monitored with thermal imaging, and pre‐ and post‐treatment leaf samples were collected from warmed and control branches for fungal DNA extraction, ITS sequencing, and community analysis. Our results show that fungal leaf endophyte richness and composition were strongly determined by host plant identity, but were unaffected by warming. Linear mixed‐effects models and paired *t*‐tests showed no significant changes in ESV richness with heating across all hosts. Similarly, distance‐based redundancy analysis and PERMANOVA tests revealed no significant shifts in fungal community composition due to increased leaf temperatures. However, a random forest classification model identified a subset of fungal ESV that were predictive of temperature treatment. While overall endophyte communities were stable, specific taxa may be thermally responsive. Foliar fungal endophyte communities in subtropical woody plants may be resistant to short‐term warming. Such stability could buffer host plants against microbial disruption during temperature extremes, though it may also limit adaptive microbial turnover. This study advances understanding of plant–microbe thermal dynamics and underscores the need for further research on the functional resistance of endophyte communities under climate change.

## Graphical abstract

Fungal endophytes play important roles in plant ecology, yet their responses to leaf‐level warming remain poorly understood. We experimentally increased leaf temperatures in six native subtropical plant species and found that endophyte richness and community composition were strongly structured by host identity but largely unaffected by short‐term warming. These results suggest that foliar fungal endophyte communities may be resilient to these increases in leaf temperature.

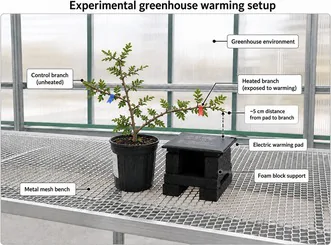

## 1 Background

Microbiomes play many essential roles in ecosystem functioning, including through beneficial effects on plant growth, survival, and fecundity. Fungal endophytes, which occur in all plant lineages and inside all compartments of plants, can determine growth and defense of their hosts and may contribute to plant health and the extreme biodiversity of tropical and subtropical ecosystems (Gilbert and Strong 2007; Rodriguez et al. 2008). Their interactions with hosts span a continuum from mutualistic to neutral or even latent pathogenic, depending on the host, microbial taxon, and environmental conditions. Foliar endophytes can increase abiotic and biotic stress tolerance of hosts through changes in morphology and investment (e.g., root structure and investment in roots versus shoots), physiological attributes (e.g., stomatal conductance), and defensive characteristics (e.g., production of anti‐herbivore alkaloids; Rodriguez et al. 2009). However, while foliar endophytes are critical to plant productivity and survival, we know surprisingly little about how these fungal communities will respond to rapid warming. Although several studies have investigated the effects of experimental warming on foliar endophyte communities, most have manipulated ambient air or whole‐plant temperatures. In contrast, relatively little is known about how changes in leaf temperature (the microenvironment directly experienced by foliar endophytes) influence these communities, particularly in tropical and subtropical tree species. Moreover, how increasing temperatures will impact foliar endophytes and the services that they provide their plant hosts is especially understudied in tropical and subtropical plant species (González‐Teuber et al. 2019; Van Bael et al. 2016). The limited understanding of how climate change affects foliar endophytes in tropical and subtropical plants is deeply concerning, as these regions represent important hotspots of global plant biodiversity. Among the environmental factors that influence endophyte communities, temperature has emerged as an important driver. Studies have shown that climatic variables like temperature and precipitation can explain variation in endophyte richness and composition (Zimmerman and Vitousek 2012). Historical precipitation and temperature together explain approximately 68% of the variation in fungal endophyte richness, exceeding the contribution of short‐term annual weather conditions (Giauque and Hawkes 2016). However, these studies largely focus on air temperatures, overlooking the more proximate conditions that fungi experience within the plants and leaves itself (i.e., the fungal microclimate).

Importantly, leaf temperatures can differ dramatically from surrounding air temperatures due to a combination of thermoregulatory behaviors, morphology, and physical traits of the leaves (Lin et al. 2017; Leigh et al. 2017; Tserej and Feeley 2021). For example, leaf size, color, and orientation can cause co‐occurring plants to vary in leaf temperature by as much as 11°C, even under identical air temperatures (Fauset et al. 2018). Moreover, within a single plant, sun and shade leaves may differ significantly in temperature, with sun leaves often exhibiting traits that promote heat dissipation (Smith and Nobel 1977).

Given that fungi colonize and persist within the leaf microenvironment, leaf temperature should play a strong role in shaping endophyte communities. Although this relationship has not been directly tested, several studies support this hypothesis indirectly. For example, leaf color, which has been connected with leaf temperatures (Singh 1978), has also been associated with endophyte community assembly (Van Bael et al. 2016). Likewise, other functional traits related to leaf temperatures, such as leaf mass per area (LMA), leaf thickness, and chlorophyll content (Leigh et al. 2012; Sanchez‐Azofeifa et al. 2011), have also been associated with fungal endophyte hyphal growth (Nezhad and Geitmann 2013), endophyte abundances (Tellez et al. 2022), and endophyte species richness (Talebi 2011). For example, endophytes were less abundant and less diverse in thick, mechanically resistant leaves than in thinner, softer leaves, and endophyte richness decreased with increasing LMA, leaf punch strength, and carbon content (Tellez et al. 2022). Finally, total terpenoid content, which quantifies terpenoid metabolites that significantly increase during heat shock treatments (Copolovici et al. 2012), has been positively correlated with fungal endophyte frequency (González‐Teuber et al. 2019). Taken together, this evidence all suggests that endophytic community properties like richness and composition should be associated with leaf temperatures and that changes in leaf temperatures should drive changes in endophyte communities. In the context of global climate change, understanding how leaf temperature influences endophyte communities is increasingly urgent. As anthropogenic warming continues, global air temperatures, and consequently leaf temperatures, are expected to rise significantly (Seneviratne et al. 2021; Calvin et al. 2023). Elevated leaf temperatures may act as an environmental filter by favoring thermotolerant taxa while limiting the establishment, growth, or persistence of temperature‐sensitive fungi. Such filtering could reduce endophyte richness and alter community composition if only a subset of the original fungal community can tolerate warmer leaf conditions. These shifts may have potential downstream effects on plant health, community dynamics, and ecosystem function. Because there is currently no established framework linking individual foliar endophyte taxa to leaf‐temperature sensitivity, we did not formulate a priori predictions for specific taxa. Instead, our taxon‐level analyses were exploratory and aimed to identify candidate taxa whose abundance patterns were associated with experimental leaf warming. These analyses provide a basis for future work linking endophyte taxonomy and functional traits with leaf thermal microclimates.

Here, we present a novel experimental test of the hypothesis that increased leaf temperatures alter fungal endophyte communities. We manipulated leaf temperature directly in situ by experimentally heating leaves of six native Florida woody plant species and compared fungal endophyte diversity and composition between two warming treatments (control and warmed) at two sampling time points (pre‐treatment and post‐treatment). These comparisons were specifically designed to test the hypothesis that increased leaf temperatures will affect fungal endophyte richness and composition and the prediction that warmed leaves would host a less diverse and compositionally distinct fungal endophyte community.

## 2 Methods

### 2.1 Study Species

Six native Florida plant species were selected for this study (Table 1). Six individuals of each species were obtained from the Fairchild Tropical Botanic Garden and commercial nurseries in Miami‐Dade county (FL, USA) and maintained in the University of Miami's greenhouse (Coral Gables, FL USA; 25°43′25.59″ N, 80°16′48.87″ W). At the start of the experiment, all plants were < 1 year old and were grown in 1‐ or 3‐gal nursery pots containing the standard commercial potting substrate used by the supplying nurseries. During the experiment, greenhouse conditions averaged 27.1°C ± 1.4°C and 66.9% ± 10.3% relative humidity under natural daylight conditions.

**TABLE 1:** Study species and environmental tolerances.

Species	Common name	Light preference	Temperature tolerance	Drought tolerance	Soil preference	Citations
*Hamelia patens*	Firebush	Full sun to partial shade	Moderate	Moderate	High pH (alkaline) soils	Gilman and Meerow (1999)
*Conocarpus erectus*	Buttonwood	Full sun	High	High	Well‐drained, saline soils	Rehman et al. (2019)
*Ernodea littoralis*	Golden creeper	Full sun	High	High	Well‐drained, sandy	Francis (2004)
*Psychotria nervosa*	Wild Coffee	Partial shade	Low–Moderate	Low–Moderate	Well‐drained, non‐saline	FNPS (2024b)
*Myrcianthes fragrans*	Simpson's stopper	Full sun to partial shade	High	High	Calcareous, sandy, rocky soils	FNPS (2024a)
*Guaiacum sanctum*	Tree of life	Full sun to partial shade	High	Very high	Clay, sand, loam, wide pH range	Gilman et al. (2022)

### 2.2 Warming Experiment

To investigate the expected effects of leaf temperature on endophyte communities, we experimentally increased leaf temperatures of individual branches using 15 × 20 cm electric warming pads that maintain constant temperatures of 40°C–50°C (Figure 1). For each individual plant, one branch was assigned to the warmed treatment and one separate branch on the same plant was assigned to the unheated control treatment. The heaters were placed approximately 5 cm below one branch of six individuals per species. A pilot comparison of leaf temperatures in heated and non‐heated leaves showed that this treatment warmed the leaves by an average of 1°C–3°C compared to leaves on non‐treated branches of the same individual plants and consistent temperatures were established after 3 days.

**FIGURE 1:**
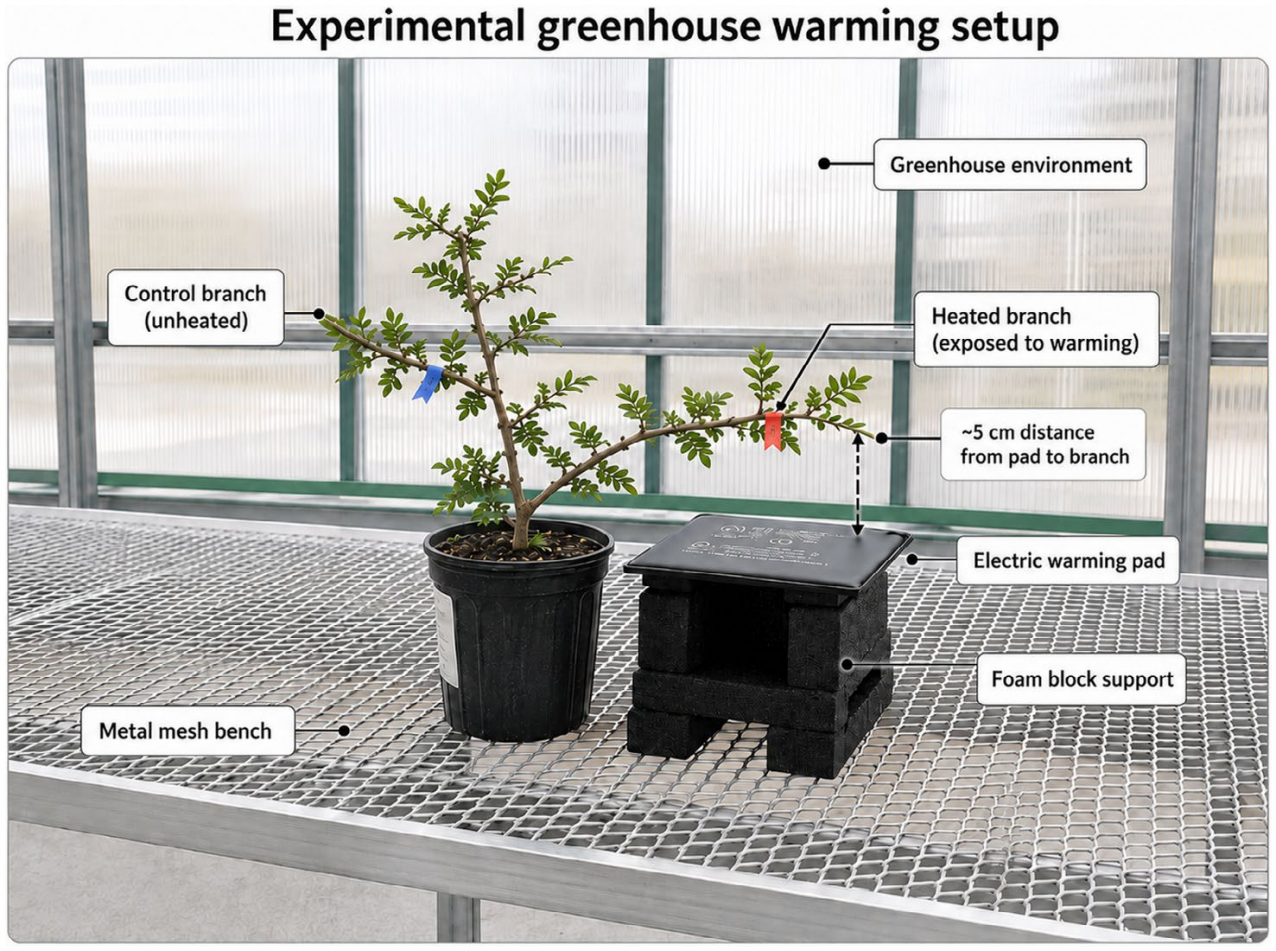
Experimental warming setup.

### 2.3 Leaf Thermal Measurements

Prior to the experiment, we collected six leaves each from one warmed and one control branch per individual for fungal DNA extraction to determine pre‐treatment endophyte composition. After the start of treatment, we took thermal measurements on another set of 12 leaves (6 heated and 6 unheated control leaves) of each individual plant. Leaf temperature measurements were conducted 3 times per week using a thermal camera (FLIR E95, thermal sensitivity and emissivity set at 0.95). Once consistent leaf temperature differences were established between the groups (warmed vs. control) for a period of 2 weeks, the leaves were harvested (post‐treatment leaves) and brought to a laboratory facility for fungal DNA extraction. The leaves were frozen immediately after harvest and stored at −80°C until DNA extraction.

### 2.4 Fungal DNA Extraction and Sequencing

DNA was extracted from 144 leaf samples representing six plant species, six individuals per species, two warming treatments (control and warmed), and two sampling time points (pre‐treatment and post‐treatment): 6 species × 6 individuals × 2 warming treatments × 2 sampling time points using a Qiagen DNeasy Plant Pro Kit (catalog number: 69206). For our extraction protocol, we first homogenized frozen leaf tissue in a TissueLyser (Qiagen TissueLyser II Bead Mill) for 5 min at 25 Hz with blocks pre‐frozen at −20°C and with 1.58 mm (1/16 in) stainless steel beads. This homogenization step pulverizes the leaf tissue to improve the subsequent physical and chemical lysis steps. We then lysed pulverized leaf tissue in a cetyltrimethylammonium bromide solution (CTAB; F2 + K buffer [70% of F2 buffer, 29.99% of 5 M NaOAc pH 5.2, 0.01% of β‐mercaptoethanol]; F2 buffer [20% of 10% CTAB, 10% of 1 M Tris–HCl pH 8, 10% of 20% PVP40, 4% of 0.5 M EDTA pH 8.0, 50% of 4 M NaCl, 6% of H_2_O]) by incubating resuspended tissue at 65°C for 15 min then homogenizing in a TissueLyser for 2 min at 25 Hz. We repeated the warming and homogenization steps four times to improve lysis due to characteristics like thick cuticle layers that made these species difficult to lyse. After this physical/chemical lysis in CTAB solution, we then followed the standard DNeasy Plant Pro Kit protocol for DNA binding and subsequent steps.

All negative controls had undetected fluorometer (Qubit 4; Qubit High Sensitivity DNA Assay, Q33231) readings indicating contaminants were not present during the extraction process. Sequencing primers (Revillini et al. 2022) were used that matched the universal tail sequences from the first round of amplification. Fungal DNA was targeted using primer pairs ITS1/ITS2 for PCR (White et al. 1990). Libraries were prepared for sequencing using a two‐step dual indexing protocol (Gohl et al. 2016). After each PCR step, magnetic bead cleaning was performed and DNA quality was checked using 1% agarose gel electrophoresis. Indexed DNA from 144 samples was pooled in equimolar quantities. Then, libraries were sequenced on an Illumina MiSeq Sequencer (v3, 300 bp paired end) at the University of Miami's Center for Genome Technology (Miami, FL, USA).

### 2.5 Bioinformatics

Sequences were processed through QIIME2 (v.2023.9) to join paired‐end reads, remove low quality bases, and classify reads into Exact Sequence Variants (ESVs) (Bolyen et al. 2019). Denoising was performed with the DADA2 algorithm (Callahan et al. 2016), to remove chimeric sequences and truncate amplicon forward and reverse reads to an equal length. We determined taxonomy using the UNITE (v_28.07.2023) fungal database (Koljalg et al. 2020) which determines “species” based on 99% similarity thresholds of the ITS1 region. We then used the UNITE database to build a Bayesian, supervised machine learning approach for matching our reads to the UNITE database. We first built a classifier of the UNITE database (fit‐classifier‐naive‐bayes function) which builds Bayesian models for each reference sequence in the database to determine the probability that a read belongs to the same “species” as the reference. We then used that classifier to match reads to the UNITE database (classifier‐sklearn function). All relative abundance tables from QIIME2 were imported into R v4.1.173 (R Core Team 2023). Replicates were pooled and rarefied to 2000 reads based on rarefaction curve analysis (R package GuniFrac, v1.5) (Chen et al. 2018) and 21 samples were eliminated from the analysis. Following best practices (Reitmeier et al. 2021), sequences with relative abundances of < 0.25% were removed to avoid overinflating diversity due to spurious sequences. Exclusions occurred across all four warming‐treatment × sampling‐time combinations and across five of the six host species rather than being restricted to a single experimental group.

### 2.6 Statistical Analysis

To assess the relationships between endophyte fungal ESV richness of warmed vs. control leaves collected from individual plants pre‐ and post‐treatment, we used a global linear mixed‐effects model using the *nlme* package in R (Pinheiro et al. 2012). Warming treatment, pre‐post effect, and plant species, as well as their interactions were included as fixed effects, and individuals were included as random effects (random intercepts and slopes). We also assessed how the warming treatment affected the composition of the fungal endophytes. We used Bray–Curtis distances to quantify dissimilarities in ESV composition between warmed and control leaves. We then performed a distance‐based redundancy analysis to assess response to time, treatment, and plant species and a PERMANOVA to test for factor significance (999 permutations). We tested branch‐level pre‐to‐post richness change against mean leaf temperature using a mixed‐effects model with random intercepts for species and individual plants nested within species. For community composition, we used a species‐conditioned permutation analysis that preserved plant pairing.

To identify fungal ESVs whose abundance patterns contributed to distinguishing warmed from control samples, we conducted an exploratory random forest classification analysis with Boruta feature selection using the Boruta package in R (Kursa and Rudnicki 2020). The predictor matrix consisted of rarefied ESV counts, with samples represented by rows and ESVs by columns. Sequence counts were rarefied to 2000 reads per sample and filtered to remove low‐abundance sequences prior to analysis. Warming treatment, classified as control or warmed, was used as the response variable. Boruta evaluates predictor importance by comparing each ESV with randomized shadow features and retains those that consistently contribute more strongly to classification than the best‐performing shadow feature. The analysis was used to identify ESVs that were informative for distinguishing warming treatments. Because random forest classification does not provide a direct test of differential abundance, the selected ESVs were interpreted as candidate taxa associated with treatment classification.

## 3 Results

### 3.1 Diversity

A comparison of daytime leaf temperatures between warmed and control leaves showed consistent temperature increases, with a few isolated exceptions in *Hamelia patens* . Differences in mean temperatures of control and warmed leaves were 2.1°C (±1.2°C) and ranged from 0.1°C in *H. patens* to 5.4°C in *Myrcianthes fragrans* (Figure 2). *Hamelia patens* leaves likely resisted warming because of high transpiration and morphological adaptations that allowed them to maintain stable temperatures despite external heat input (Tserej and Feeley 2021).

**FIGURE 2:**
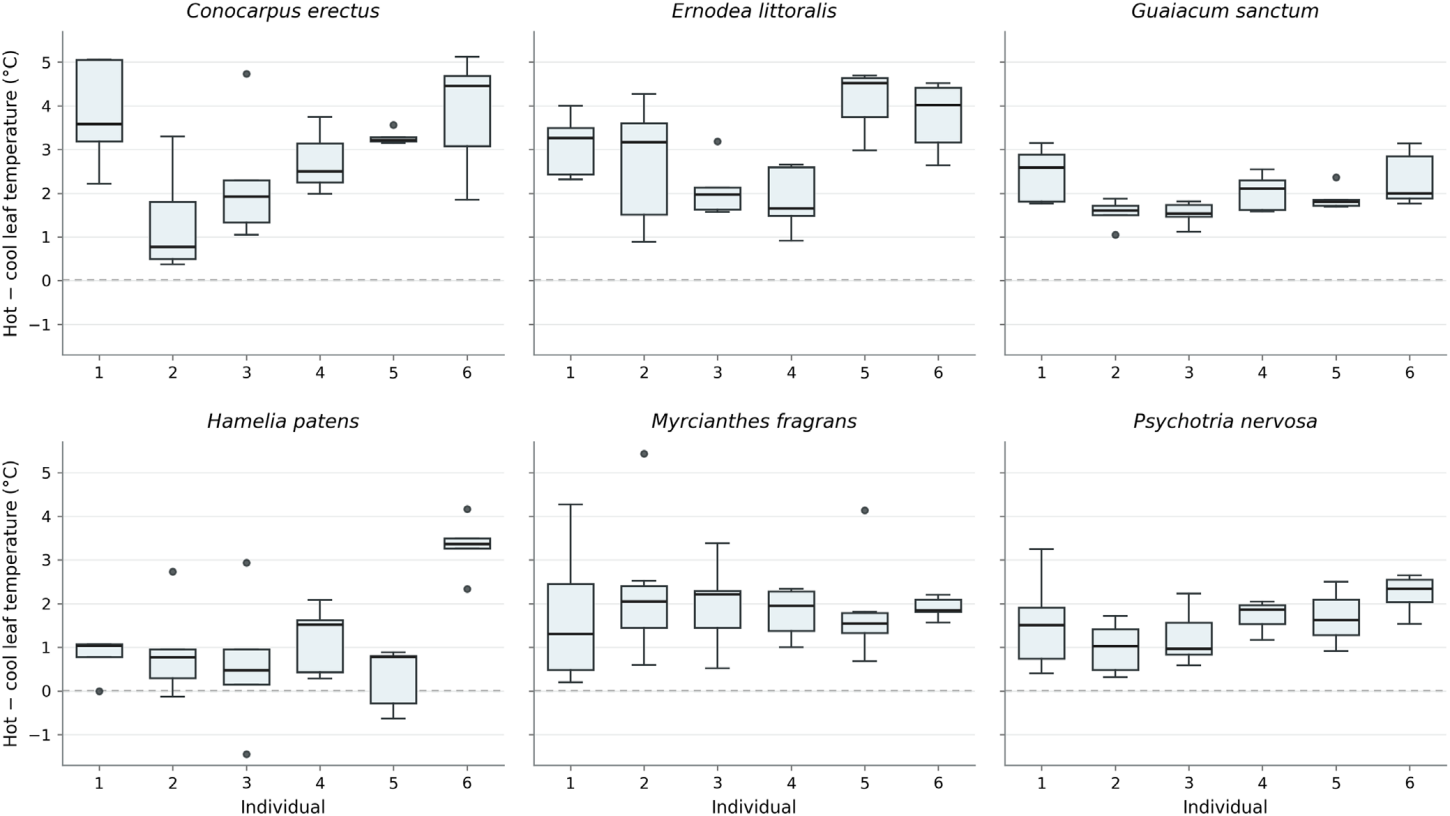
Variation in leaf temperature difference between heated and non‐heated leaves over the course of 2 weeks for each individual of each species.

Endophyte ESV richness was primarily determined by host species identity (*p* = 0.001) and marginally by the interaction between host species identity and time (meaning pre vs. post samples, *p* = 0.05). Warming had no effect on ESV richness (*p* = 0.88; Table 2). For each plant species, we tested whether fungal endophyte richness changed from the pre‐treatment to the post‐treatment sampling period separately for control branches and heated branches. Therefore, each plant species had two paired *t*‐tests and two corresponding *p*‐values: one testing the change in richness from pre‐ to post‐treatment in control branches, and one testing the change in richness from pre‐ to post‐treatment in heated branches. Paired *t*‐tests also found no significant effects of heating on endophyte richness in any of the six plant species (Figure 3; Table 3). The continuous‐temperature analysis also found no detectable association between mean leaf temperature and community change (Bray–Curtis permutation *p* = 0.182; Hellinger sensitivity *p* = 0.077).

**TABLE 2:** Effects of experimental leaf warming, sampling time, and host plant species on fungal endophyte richness.

Fixed effect	Numerator df	Denominator df	*F*	*p*
Warming treatment	1	69	0.023	0.88
Sampling time	1	69	0.609	0.438
Host plant species	5	30	5.332	0.001
Warming treatment × Sampling time	1	69	0.02	0.888
Warming treatment × Host plant species	5	69	0.789	0.561
Sampling time × Host plant species	5	69	2.304	0.054
Warming treatment × Sampling time × Host plant species	5	69	0.395	0.851

**FIGURE 3:**
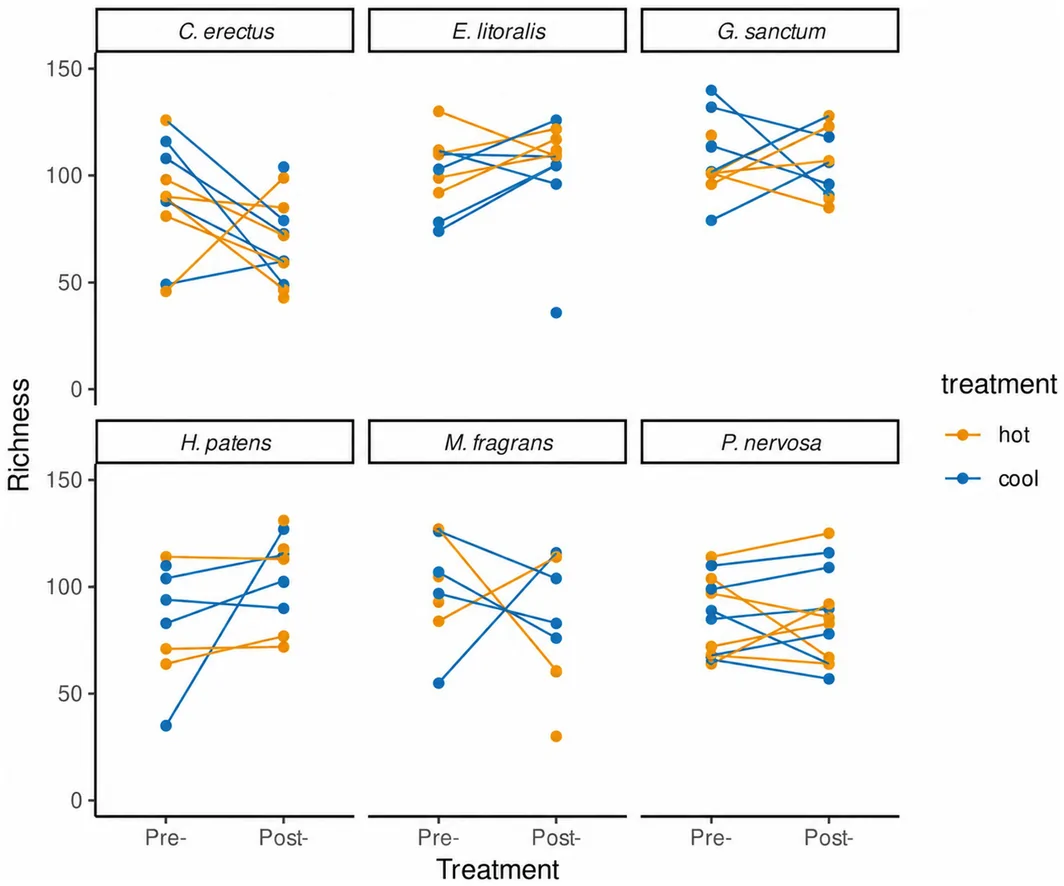
Pairwise comparisons illustrating richness differences between pre‐ and post‐treatment in unheated and heated leaves. Each line represents one individual. Each box represents a species. C: *Conocarpus erectus* , E: *Ernodea littoralis* , H: *Hamelia patens* , G: *Guaiacum sanctum* , M: *Myrcianthes fragrans* , and P: *Psychotria nervosa* .

**TABLE 3:** Paired *t*‐tests of changes in fungal endophyte richness from pre‐ to post‐treatment sampling within control and heated branches of each host plant species.

Species	Branch treatment	Mean difference	*t*	df	*p*	95% CI lower	95% CI upper
*Conocarpus erectus*	Cool	33.2	2.58	4	0.062	−26.1	92.5
*Conocarpus erectus*	Heated	8.4	0.511	4	0.636	−67.3	84.1
*Ernodea littoralis*	Cool	−12.8	−1.41	4	0.232	−54.7	29.1
*Ernodea littoralis*	Heated	−6.75	−0.690	3	0.54	−63.9	50.4
*Hamelia patens*	Cool	−29.8	−1.39	3	0.257	−154	94.8
*Hamelia patens*	Heated	−4.33	−0.991	2	0.426	−47.7	39.1
*Guaiacum sanctum*	Cool	5.6	0.388	4	0.718	−60.9	72.1
*Guaiacum sanctum*	Heated	−11.0	−1.07	3	0.363	−71.0	49
*Myrcianthes fragrans*	Cool	1.5	0.071	3	0.948	−122	125
*Myrcianthes fragrans*	Heated	18	0.375	1	0.772	−3038	3074
*Psychotria nervosa*	Cool	0.5	0.088	5	0.933	−22.4	23.4
*Psychotria nervosa*	Heated	0.333	0.036	5	0.972	−36.7	37.4

### 3.2 Composition

Plant species identity was the main factor determining the fungal endophyte composition, explaining approximately 33% of the variation in endophyte community composition (db‐RDA, *p* = 0.001). However, there were no significant differences in fungal community composition due to the warming treatment (Table 4, Figure 4). The resistance of fungal composition to warming was seen in all plant species regardless of how dissimilar the communities are between plant species. For instance, *Psychotria nervosa* and *H. patens* had the most different fungal communities from all other plant species, with community composition of foliar endophytes in these two species just as resilient to heating as the communities in *Conocarpus erectus, Ernodea littoralis, Guaiacum sanctum*, and *Myrcianthes fragrans* . The continuous‐temperature analysis also found no detectable association between mean leaf temperature and community change (Bray–Curtis permutation *p* = 0.182; Hellinger sensitivity *p* = 0.077).

**TABLE 4:** PERMANOVA results for post‐treatment fungal endophyte community composition.

Predictor	df	Sum of squares	*R* ^2^	*F*	*p*
Host plant species	5	5.801	0.354	6.365	0.001
Warming treatment	1	0.209	0.013	1.147	0.274
Residual	57	10.39	0.634	—	—
Total	63	16.4	1	—	—

**FIGURE 4:**
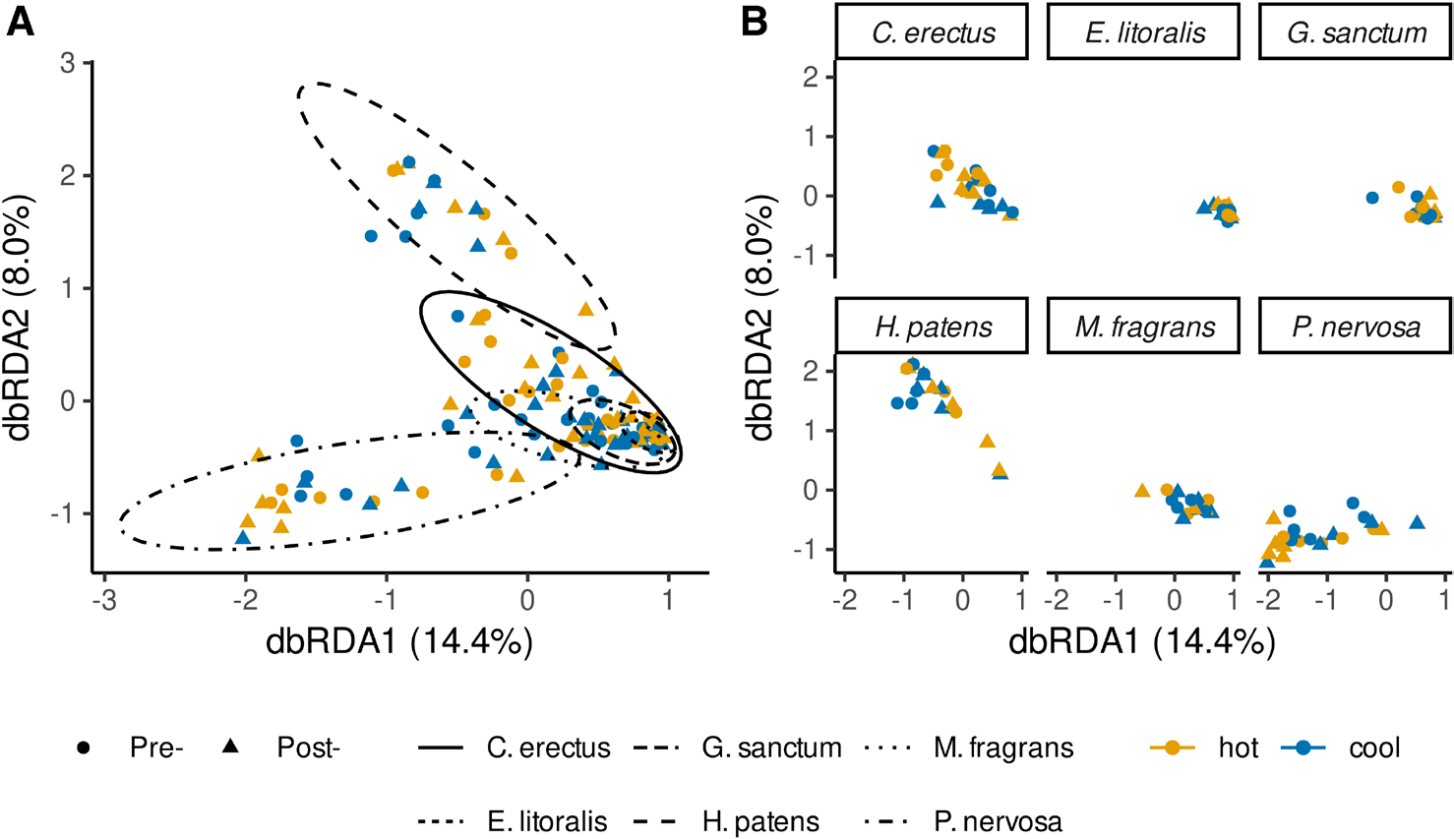
Distance‐based redundancy analysis of community composition of fungal communities post‐treatment in (A) all plant species, and (B) in individual plant species (C: *Conocarpus erectus* , E: *Ernodea littoralis*, H: *Hamelia patens* , G: *Guaiacum sanctum* , M: *Myrcianthes fragrans* and P: *Psychotria nervosa* ). The ellipses around data points show the 95% confidence interval for each species. The plots in B are the same points from A, but filtered to only display the data points for that species.

### 3.3 Random Forest Analysis

The random forest analysis identified three ESVs whose abundance patterns were predictive of temperature treatments. These included two Basidiomycota, a fungal ESV from the class *Cystobasidiomycetes* and *Kondoa* sp. from the class Agaricostilbomycetes as well as an Ascomycota—*Zasmidium citrigriseum* (class Dothideomycetes). Two of these ESVs (*Kondoa* sp. and *Zasmidium citrigriseum*) had lower mean rarefied counts in warmed samples, whereas one (the Basidiomycota ESV belonging to the class Cystobasidiomycetes) had higher mean rarefied counts in warmed samples. These patterns were descriptive and were not direct tests of differential abundance. The random forest classifier had an out‐of‐bag (OOB) error rate of 29.7%, corresponding to an OOB classification accuracy of 70.3%. Classification performance was similar between treatments, with class‐specific error rates of 30.3% for control samples and 29.0% for warmed samples.

## 4 Discussion

Our results showed that experimental warming increased leaf temperatures by an average of 2.1°C but did not significantly affect fungal endophyte richness or community composition across the six focal tree species. Instead, host plant identity was the primary driver of endophyte community structure, while a small number of individual taxa showed responses to warming. These findings suggest that subtropical leaf fungal endophyte communities may be resistant to short‐term temperature increases. The continuous‐temperature analyses were consistent with this overall pattern: we found no clear association between mean leaf temperature and richness or community change. The richness estimate was imprecise, so this result should not be interpreted as evidence that temperature has no effect.

Fungal endophytes have important influences on plant performance and plant environmental tolerances and changes in fungal endophytes may be one mechanism through which plants can indirectly acclimate to environmental changes. As such, an important consideration that will influence how resilient or threatened plant communities are to climate change is the effect of rising temperatures on the diversity and composition of fungal endophytes within individual plants. Indeed, climate change can disrupt plant–microbe interactions through altered context dependency, temporal mismatches, and spatial mismatches (Rudgers et al. 2020), suggesting that shifts in endophyte communities may represent a key pathway by which warming influences plant responses to environmental stress.

Our experimental warming treatments did not lead to any consistent or significant changes in the richness or composition of leaf fungal endophytes within individuals of our six focal tree species, indicating that these symbiotic relationships may be resistant to spikes in temperature. While endophyte communities were stable across warming treatments, plant species identity accounted for 1/3 of the variation in endophyte community composition among leaves in our study. Some endophyte communities appeared partially nested within those of other hosts, potentially reflecting phylogenetic relatedness. Three focal species ( *Hamelia patens* , *Psychotria nervosa* , and *Ernodea littoralis* ) belong to Rubiaceae, and closely related hosts may share leaf traits that filter similar fungal taxa. However, our sampling design included too few host species, and too few independent representatives within most plant families, to test for a phylogenetic signal independently of host‐species identity or functional traits. Previous studies have produced mixed results. Host phylogeny explained a portion of foliar endophyte community variation among *Ficus* species (Liu et al. 2019), whereas other common‐garden and multihost studies found little or no relationship between host phylogenetic distance and endophyte community similarity (Whitaker et al. 2020). Our result emphasizes the importance of host plant identity in endophyte communities of subtropical plants, which is consistent with findings in other ecosystems (Laforest‐Lapointe et al. 2016; Hernandez et al. 2023; Rawstern et al. 2023). These results highlight several emerging patterns in the burgeoning literature on how climate warming affects endophytic microbes, such as plant species‐specific endophyte communities can be stable in the face of warming conditions and that this stability may be common across time scales, ecosystems, plant compartments, and experimental designs. For instance, at the end of a long‐term (23‐year) field experiment in a permanent grassland ecosystem that warmed plots ~2°C, Kazenel et al. (2019) found there were no significant effects of warming on leaf endophyte diversity and community composition across three species of perennial grasses. In terms of plant compartments, previous studies of root fungal endophytes have exhibited similar trends to our foliar endophyte study, where the overall diversity and composition was not affected by experimental warming (Lyons et al. 2021), and plant species identity had a strong influence on the fungal community composition. Similarly, in another experimental warming study where open‐top warming chambers were used to elevate air temperatures by 1°C–4°C, site and soil characteristics, rather than temperature, were the main factors affecting root fungal community composition in plants (Fujimura et al. 2008). Conversely, Edwards et al. (2025) found that warming reduced colonization by septate fungi by 90% in leaves and 35% in roots, and also decreased fungal diversity and altered community composition; however, their findings were based on grassland warming experiments in the United States that spanned 2–25 years across a 2000‐km gradient.

The absence of a detectable effect of leaf temperatures on endophyte diversity and composition suggests that leaf fungal endophyte communities may be resistant to short‐term warming. Furthermore, this thermal resistance in endophyte communities can be important for host plants' success. For instance, previous research has shown that seedlings inoculated with thermotolerant endophytes show higher survival rates and higher shoot/root growth under high temperatures when compared to untreated seedlings (Sangamesh et al. 2017). Moreover, fungal endophytes have been proven to help in the establishment of plants to high‐stress habitats by conferring them with a high heat tolerance (Rodriguez et al. 2008). This resistance could have positive implications for the fitness of native plant species through stable host‐symbiont associations under future climate change scenarios.

However, community resistance to warming does not necessarily indicate that no microbial change is beneficial for plant hosts. While maintaining stable endophyte communities may allow plants to preserve existing beneficial interactions during short‐term thermal stress events, long‐term increases in temperature may favor shifts toward more thermotolerant endophyte taxa. Indeed, inoculation studies have shown that thermotolerant endophytes can enhance plant survival and growth under high‐temperature conditions (Sangamesh et al. 2017). Therefore, resistance to short‐term warming may provide stability during extreme heat events, whereas longer‐term climate adaptation may require greater flexibility in endophyte community composition. Determining whether and how endophyte communities shift toward thermally adapted taxa will require long‐term warming studies.

However, while the overall community appears resilient to elevated temperatures, results from the random forest analysis indicate that individual endophyte taxa may still respond to thermal variation, implicating warming in ecological filtering or shifts in symbiotic dynamics of particularly thermal‐tolerant or susceptible taxa.

Although the ecological roles of these ESVs in our focal host species remain unknown, their taxonomic identities provide some context for interpreting their responses. *Zasmidium citrigriseum*, which decreased in abundance in warmed samples, belongs to the Mycosphaerellaceae and is best known as the causal agent of citrus greasy spot. However, *Z. citrigriseum* has also been detected as part of the endophytic fungal community of asymptomatic citrus leaves, suggesting that it can persist as a latent component of the leaf mycobiome (Huang et al. 2024). Thus, its decline with warming could represent temperature‐mediated filtering of a potentially latent pathogenic or commensal member of the endophyte community, although its ecological role in our focal hosts remains unknown.

*Kondoa* sp. also decreased in warmed samples. *Kondoa* is a genus of basidiomycetous yeasts that has been reported from leaf‐associated fungal communities and can represent an abundant component of the phyllosphere mycobiome (Ding et al. 2022). The decline of *Kondoa* in warmed leaves may therefore reflect sensitivity to altered leaf thermal conditions, although the thermal tolerance and ecological function of the particular ESV detected in our study remain unknown.

In contrast, the ESV assigned to Cystobasidiomycetes increased under warming. Cystobasidiomycetes includes numerous yeast‐form fungi, and yeasts are common members of fungal communities associated with aerial plant tissues (Gouka et al. 2022). However, the limited taxonomic resolution of this ESV prevents us from assigning a specific ecological role or thermal strategy. Nevertheless, its contrasting response relative to the two ESVs that declined with warming is consistent with the possibility that increased leaf temperature acts as an ecological filter, favoring some members of the endophyte community while suppressing others, even in the absence of detectable whole‐community turnover. Future studies isolating these fungi and directly measuring their thermal performance and effects on host plants will be necessary to determine whether these abundance patterns correspond to differences in thermal tolerance or ecological function.

A previous study assessing the thermal tolerance of endophytic fungal isolates indicated that certain operational taxonomic units can persist unaffected at temperatures as high as 45°C (Sangamesh et al. 2017). Furthermore, endophytic fungal strains isolated from a plant species in a hot region of northwestern Australia demonstrated that the majority of the fungi tested were capable of growing at 30°C, with one species thriving at 50°C (Dastogeer et al. 2019). However, the predictability of these fungal responses to climate change remains uncertain due to the scarcity of available data (Kivlin and Rudgers 2019; Lyons et al. 2021). Nonetheless, more work is needed on this topic since loss or gain of particular taxa can have important consequences for plants and ecosystems. For instance, recent work on multidimensional environmental niches of microbes showed that specialist taxa, which are microbes with narrow niche breadths across a suite of climate axes, are more likely to serve as keystone microbes providing important ecosystem services, including beneficial plant symbiosis (Hernandez et al. 2023; Rawstern et al. 2023). Because specialist species have narrow niche breadths across many axes, including temperature, these taxa may be particularly susceptible to changing climates, and their loss could have important consequences.

The absence of a strong temperature effect on leaf fungal endophyte communities may have both positive and negative implications for plant hosts, depending on ecological context. On one hand, this stability could be advantageous, as it suggests that beneficial symbiotic associations remain intact despite thermal stress, potentially maintaining plant support functions such as growth promotion, pathogen resistance, and stress tolerance (Ismail et al. 2018). These stable relationships could help subtropical plants endure warming without disruption to microbial‐mediated benefits. However, this lack of community turnover may also be limiting. Microbial community shifts, including the acquisition of novel or more thermotolerant symbionts, have been shown to enhance host resilience in other systems (Carrell et al. 2022). If the existing endophyte community lacks sufficient functional plasticity to cope with intensifying environmental stressors, the host plant's ability to adapt or acclimate to climate change may be constrained. Thus, whether endophyte community resistance to warming is ultimately beneficial or detrimental may depend on whether current symbionts can continue to meet host physiological needs under future environmental conditions.

The within‐plant experimental design allowed us to control for variation among individual plants, including host genetic differences and environmental history. However, because warming was applied to a single branch, we cannot completely exclude the possibility that systemic plant responses influenced endophyte communities in warmed leaves. Future studies incorporating whole‐plant warming approaches or longer‐term exposure will help clarify the extent to which endophyte communities respond to sustained increases in temperature.

Our study provides novel insights into the resistance of leaf fungal endophyte communities in subtropical tree species to experimental warming. Despite marked increases in leaf temperatures, we did not observe significant changes in endophyte richness or composition across the six focal tree species. The complex relationship between fungal endophytes and their host plants, particularly in the context of global warming, highlights the need for further study of these systems in tropical and subtropical forests.

## Author Contributions

**Olga Tserej:** conceptualization (lead), data curation (lead), formal analysis (equal), funding acquisition (supporting), investigation (lead), methodology (equal), project administration (lead), resources (equal), software (equal), supervision (lead), validation (equal), visualization (equal), writing – original draft (lead), writing – review and editing (lead). **Damian J. Hernandez:** conceptualization (equal), data curation (lead), formal analysis (lead), funding acquisition (supporting), investigation (equal), methodology (lead), project administration (equal), resources (equal), software (lead), supervision (equal), validation (equal), visualization (equal), writing – original draft (equal), writing – review and editing (equal). **Amanda H. Rawstern:** conceptualization (equal), data curation (equal), formal analysis (equal), funding acquisition (supporting), investigation (equal), methodology (lead), supervision (equal), validation (equal), visualization (equal), writing – original draft (equal), writing – review and editing (equal). **Kasey N. Kiesewetter:** data curation (supporting), formal analysis (equal), investigation (equal), methodology (equal), resources (equal), software (equal), validation (equal), writing – review and editing (supporting). **Brianna K. Almeida:** investigation (supporting), methodology (equal), resources (equal), validation (supporting), writing – original draft (supporting), writing – review and editing (equal). **Michelle E. Afkhami:** conceptualization (lead), funding acquisition (lead), investigation (equal), methodology (equal), project administration (equal), resources (equal), supervision (lead), writing – original draft (equal), writing – review and editing (equal). **Kenneth J. Feeley:** conceptualization (lead), funding acquisition (lead), investigation (equal), methodology (equal), project administration (equal), resources (equal), supervision (lead), writing – original draft (equal), writing – review and editing (equal).

## Funding

This work was supported by the Smathers Endowment for Tropical Biology at the University of Miami, Coral Gables, Florida, USA. This support was provided through a university endowment.

## Conflicts of Interest

The authors declare no conflicts of interest.

## Data Availability

All data and code supporting the findings of this study are publicly available on Zenodo at https://doi.org/10.5281/zenodo.18270420. The repository includes processed fungal community data, sample metadata, and analysis scripts necessary to reproduce the results.
